# Supplementary material for: Mechanisms of mitochondrial reactive oxygen species action in bone mesenchymal cells
Source: J Biol Chem. 2025 Jul 31;301(9):110551. doi: 10.1016/j.jbc.2025.110551 (PMC12423689; doi:10.1016/j.jbc.2025.110551)
Supplement: Supporting Figures S1-S5 [file mmc1.pdf]

Supporting information

Mechanisms of mitochondrial reactive oxygen species action in bone mesenchymal cells

Md Mohsin Ali[1], Intawat Nookaew[2,4], Ana Resende-Coelho[1], Adriana Marques-Carvalho[1], Aaron Warren[1], Qiang Fu[4], Ha-Neui Kim[1,4], Charles A O'Brien[1,3,4], Maria Almeida[1,3,4*]

[1]Division of Endocrinology and Metabolism, University of Arkansas for Medical Sciences, Little Rock, AR, USA

[2]Department of Biomedical Informatics; University of Arkansas for Medical Sciences, Little Rock, AR, USA

[3]Department of Orthopedic Surgery; University of Arkansas for Medical Sciences, Little Rock, AR, USA

[4]Center for Musculoskeletal Disease Research; University of Arkansas for Medical Sciences, Little Rock, AR, USA

*Correspondence: Maria Almeida, Ph.D., 4301 W Markham Street, #587, Little Rock, Arkansas 72205, USA; Tel.: +1-501-686-7856; Fax +1-501-686-8148; Email: schullermaria@uams.edu

Includes:

Figures S1-S5

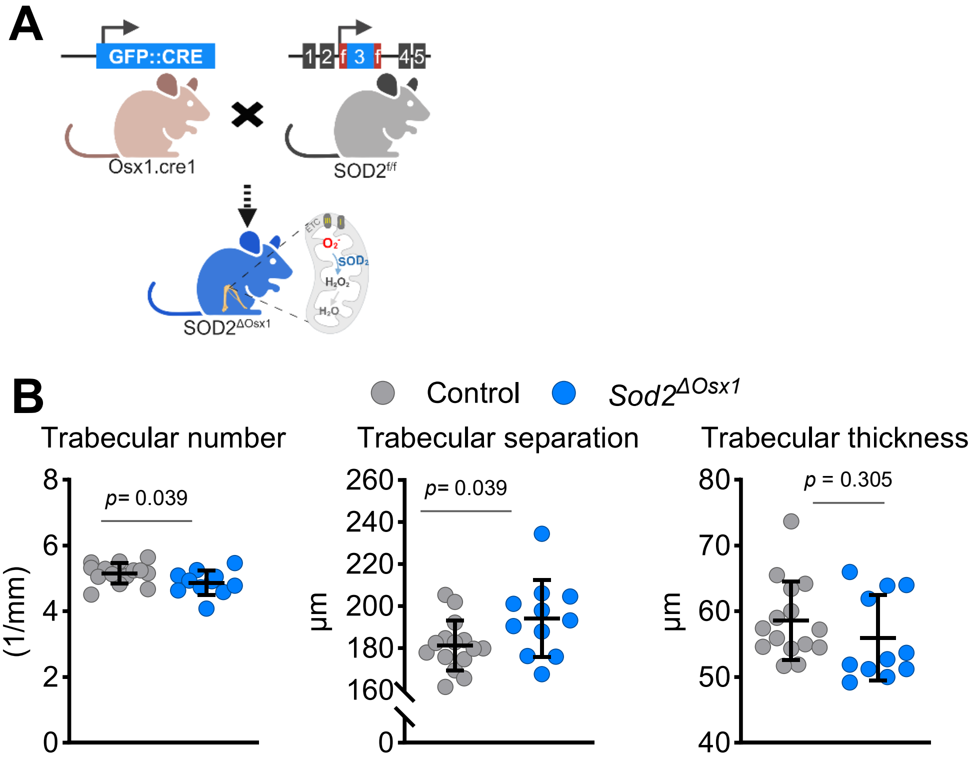

Figure S1. Breeding strategy and µCT analysis of the vertebrae from Sod2$^{\Delta Osx1}$ mice.

(A) Breeding scheme for the generation of osteoblastic lineage *Sod2* conditional knockout mice. Hemizygous Osx1-cre transgenic mice were crossed with *Sod2$^{f/f}$* mice. Resulting offspring, heterozygous for the floxed Sod2 allele with (*Sod2$^{f/+}$; Osx1-cre*) or without (*Sod2$^{f/+}$*) the Cre allele, were intercrossed to generate Sod2$^{\Delta Osx1}$ and littermate controls (*Osx1-cre*).

(B) Quantitative µCT analysis of trabecular microarchitecture in the fifth lumbar vertebra of 26-week-old male mice (n = 11-15 mice/group).

Line and error bars represent mean ± S.D. P values by two-tailed unpaired Student’s t-test. **Related to Figure 1.**

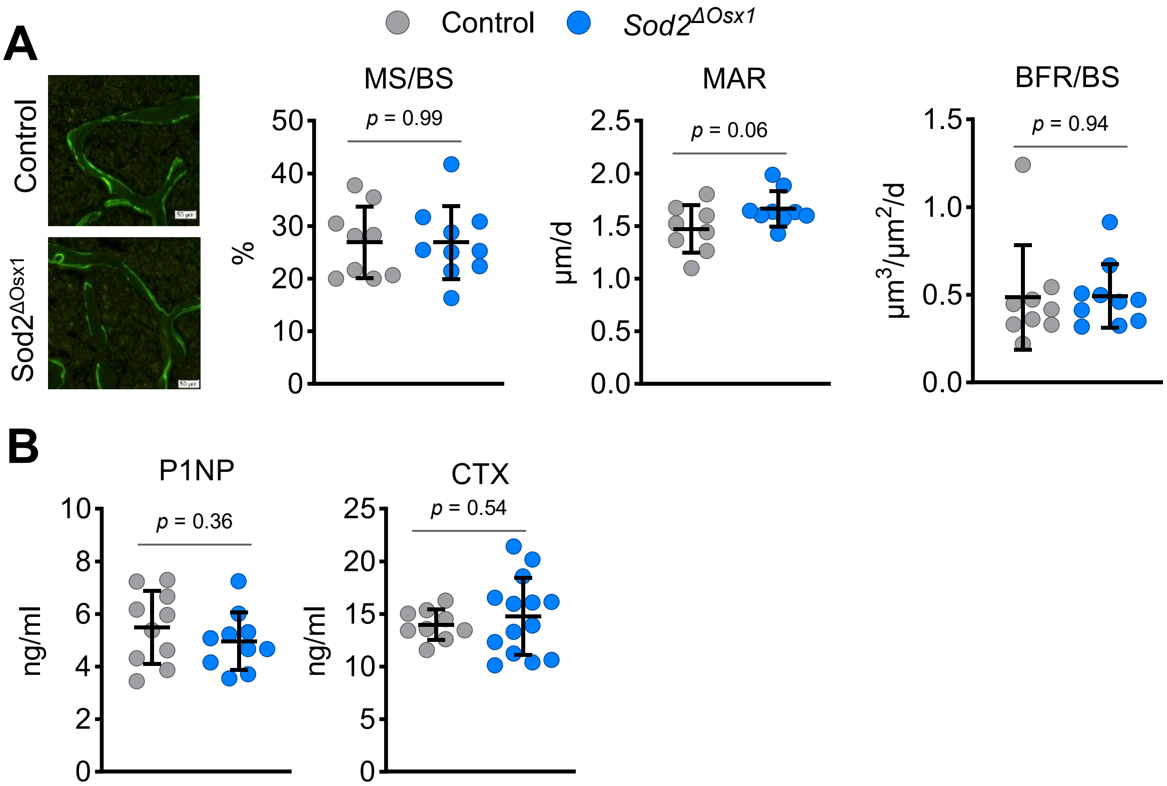

Figure S2. Histomorphometry analysis and serum bone turnover markers in 26-week-old male mice.

(A) Representative images of calcein double-labeling of trabecular bone from the 3rd lumbar vertebra, with quantification of mineralizing surface per bone surface (MS/BS), mineral apposition rate (MAR), and bone formation rate per bone surface (BFR/BS) (n = 9-10 mice/group). Scale bar: 50 µm.

(B) ELISA analysis of serum N-terminal propeptide of type I procollagen (PINP) (n = 10 mice/group), and C-terminal telopeptide of type I collagen (CTX) (n = 9-14 mice/group).

Line and error bars represent mean ± S.D. P values by a two-tailed unpaired Student's t-test. **Related to Figure 1**

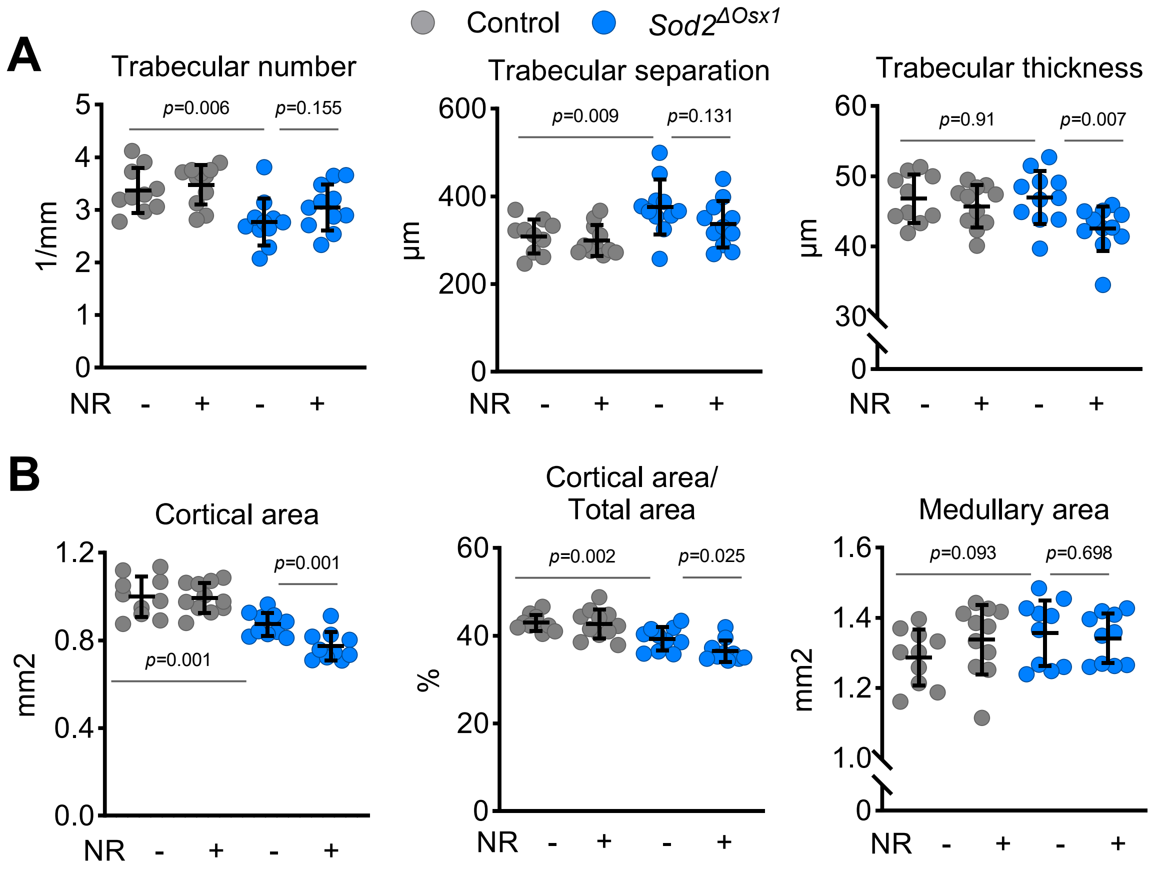

Figure S3. μCT analysis of vertebral and femoral bone from 52-week-old female Sod2$^{\Delta Osx1}$ mice supplemented with NR.

(A) Quantitative μCT analysis of trabecular microarchitecture at the fifth lumbar vertebra (n = 10-11 mice/group).
(B) Quantitative μCT analysis of cortical microarchitecture at the femur midshaft (n = 10-11 mice/group**).**

Line and error bars represent mean ± S.D. P-values by two-way ANOVA with Tukey's multiple comparisons test. **Related to Figure 3**

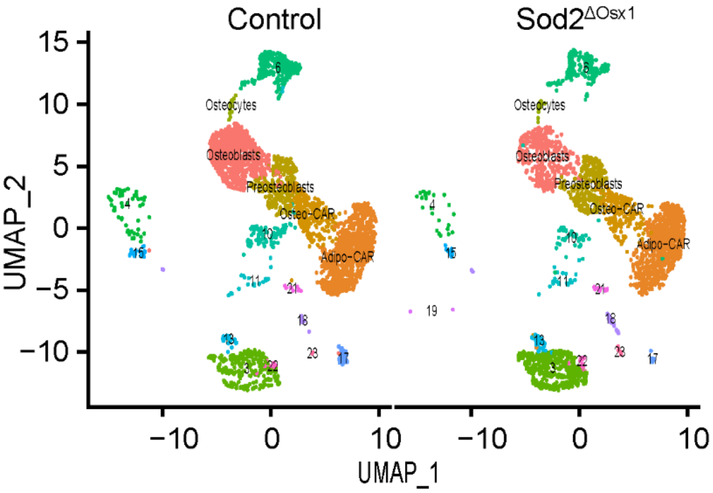

Figure S4. UMAP projection of 15,224 endosteal cells

UMAP plot of cells isolated from the endosteal compartment of 19-week-old male mice. Non-mesenchymal cell clusters are labeled numerically. **Related to Figure 4**

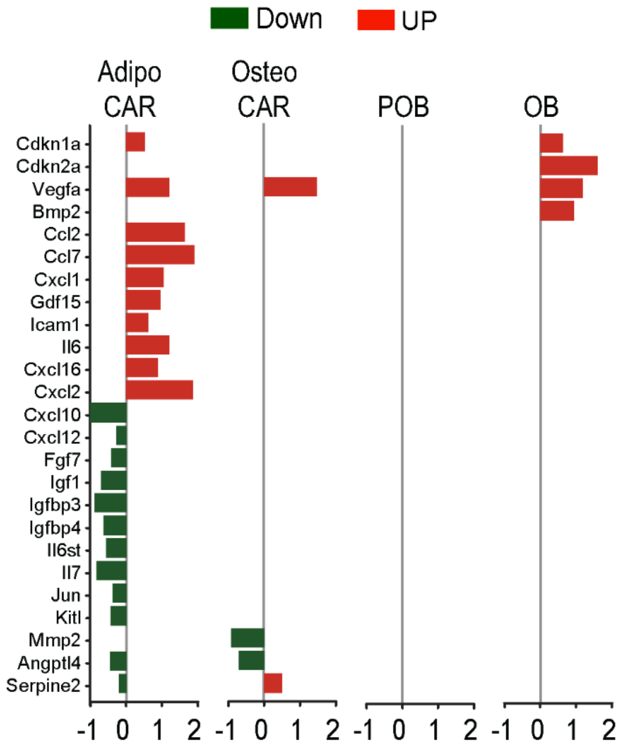

Figure S5. Differentially expressed senescence-related genes in mesenchymal cells from Sod2$^{\Delta Osx1}$ compared to control mice.

Log_2FC of differentially expressed genes between Sod2$^{\Delta Osx1}$ and control mice out of a list containing 119 senescence-related genes, curated from SenMayo (39), MSigDB (40), and CellAge (41) data sets. **Related to Figure 5**
